# The Mechanisms of Current Platinum Anticancer Drug Resistance in the Glioma

**DOI:** 10.2174/1381612828666220607105746

**Published:** 2022-08-30

**Authors:** Enzhou Lu, Ilgiz Gareev, Chao Yuan, Yanchao Liang, Jingxian Sun, Xin Chen, Ozal Beylerli, Albert Sufianov, Shiguang Zhao, Guang Yang

**Affiliations:** 1 Department of Neurosurgery, The First Affiliated Hospital of Harbin Medical University, Harbin, 150001, China;; 2 Central Research Laboratory, Bashkir State Medical University, Ufa, 450008, Russia;; 3 Department of Neurosurgery, Shenzhen University General Hospital, Shenzhen, 518055, China;; 4 Department of Neurosurgery, Sechenov First Moscow State Medical University (Sechenov University), Moscow, Russia

**Keywords:** Glioma, platinum drugs, resistance mechanisms, chemosensitivity, prognosis, microRNAs, long non-coding RNAs

## Abstract

Gliomas are the most common and malignant primary tumors of the central nervous system (CNS). Glioblastomas are the most malignant and aggressive form of primary brain tumors and account for the majority of brain tumor-related deaths. The current standard treatment for gliomas is surgical resection supplemented by postoperative chemotherapy. Platinum drugs are a class of chemotherapeutic drugs that affect the cell cycle, and the main site of action is the DNA of cells, which are common chemotherapeutic drugs in clinical practice. Chemotherapy with platinum drugs such as cisplatin, carboplatin, oxaliplatin, or a combination thereof is used to treat a variety of tumors. However, the results of gliomas chemotherapy are unsatisfactory, and resistance to platinum drugs is one of the important reasons. The resistance of gliomas to platinum drugs is the result of a combination of influencing factors. Decreased intracellular drug concentration, enhanced function of cell processing active products, enhanced repair ability of cellular DNA damage, and blockage of related apoptosis pathways play an important role in it. It is known that the pathogenic properties of glioma cells and the response of glioma towards platinum-based drugs are strongly influenced by non-coding RNAs, particularly, by microRNAs (miRNAs) and long non-coding RNAs (lncRNAs). miRNAs and lncRNAs control drug sensitivity and the development of tumor resistance towards platinum drugs. This mini-review summarizes the resistance mechanisms of gliomas to platinum drugs, as well as molecules and therapies that can improve the sensitivity of gliomas to platinum drugs.

## 1 INTRODUCTION

The platinum compound was synthesized for the first time in 1844, and then in 1965, American biologist Rosenberg accidentally discovered that cisplatin could inhibit tumor growth, which unveiled the prelude to the use of platinum drugs in tumor treatment [1]. After rigorous animal and clinical trials, the first platinum anticancer drug was approved by the United States Food and Drug Administration (US FDA) to treat testicular cancer in 1978. With the extensive development of related research around the world, platinum drugs for tumor treatment have developed to the third generation, including cisplatin, carboplatin, nedaplatin, oxaliplatin, and lobaplatin. At present, platinum drugs have been widely used in the clinical treatment of testicular tumors, ovarian tumors, lung cancer, and head and neck tumors, which have greatly improved the prognosis of these patients [2-6].

Glioma is the most common malignant tumor of the central nervous system, accounting for about 50% of neuroepithelial tumors, and is currently one of the tumors with an extremely poor prognosis in adults. In view of the infiltrative growth of gliomas to the surrounding brain tissue, it is often difficult to achieve complete resection by surgery. Therefore, postoperative adjuvant chemotherapy is of great significance in killing the remaining tumor cells, preventing tumor recurrence, and improving the survival prognosis of patients. Platinum-based anticancer drugs attack a single target, cellular DNA, and their direct coordination of nucleobases to nucleophilic nitrogen plays an important role in the induction of tumor cell apoptosis [7]. Many platinum complexes have been designed to optimize platinum-DNA interactions, and increasing their affinity for DNA reduces the exposure of platinum to other cellular nucleophiles [8]. This effect can lead to the reduction of side effects [9]. Platinum drugs are used clinically in the treatment of gliomas, but due to the existence of drug resistance, the clinical effect of chemotherapy is not satisfactory, which hinders their clinical application [10-13]. Therefore, a comprehensive understanding and study of the molecular mechanisms of platinum anticancer drug resistance are of profound significance for the development of new glioma combination therapy and the development of new platinum drugs for the treatment of gliomas.

## 2 MECHANISMS OF ANTITUMOR ACTION

Platinum drugs are antitumor drugs that act on the cell cycle. When platinum drugs enter the cytoplasm through transmembrane transport, the dissociation reaction occurs in the cell. While Cl^-^ is removed, it combines with two water molecules and finally produces hydrated platinum cations. Platinum cations may enter the cell nucleus through the nuclear pore complexes, and then combine with biological macromolecules such as protein, DNA, and RNA in the cell nucleus, of which DNA is the main target.

**Fig. (1):**
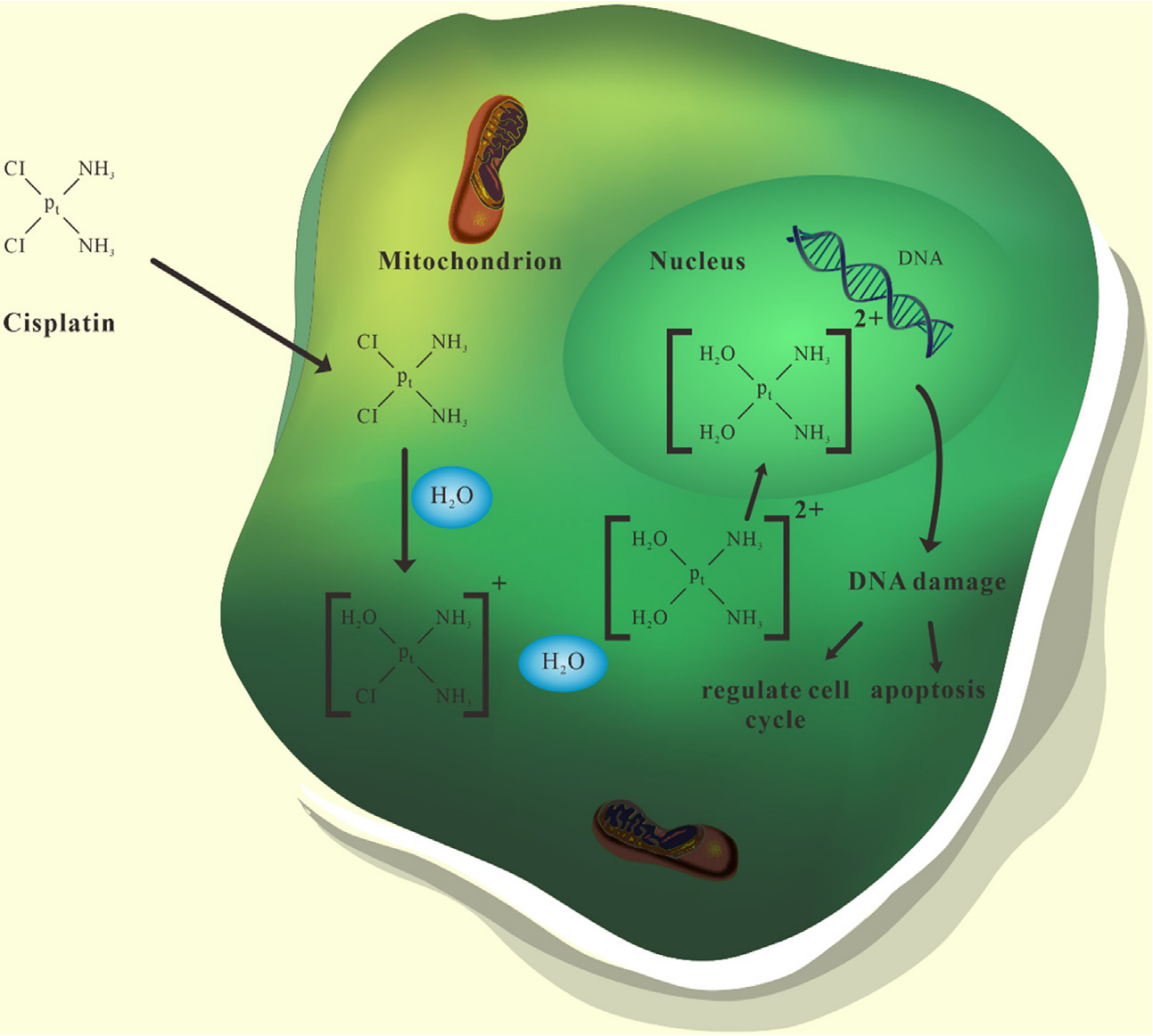
Antitumor mechanisms of platinum drugs. When platinum drugs enter the cytoplasm, they bind to two water molecules to produce hydrated platinum cations. After entering the nucleus, platinum cations can form pairing cross-links with target DNA. These cross-links will destroy the structure of DNA, thereby inhibiting DNA replication, and ultimately leading to cell cycle failure and apoptosis in tumor cells.

The position where platinum cations bind to DNA is the N7 atom of guanine and adenine. When the platinum cation is combined with the N7 atom, platinum drugs form inter-strand pairing cross-links and intra-strand pairing cross-links with target DNA. These two kinds of cross-links will destroy the local structure of duplex DNA, thereby inhibiting DNA replication, and ultimately leading to cell cycle failure and apoptosis in tumor cells (Fig. **1**) [14, 15].

## 3 MECHANISMS OF DRUG RESISTANCE

**Fig. (2):**
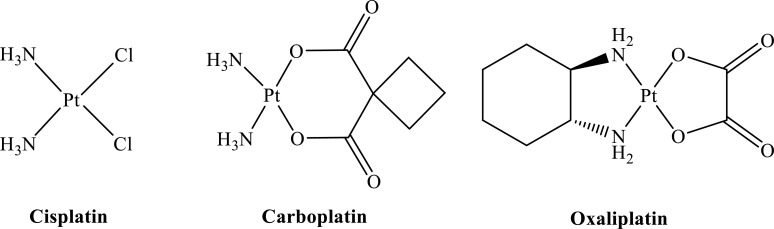
Chemical structures of the platinum drugs discussed in the review.

**Fig. (3):**
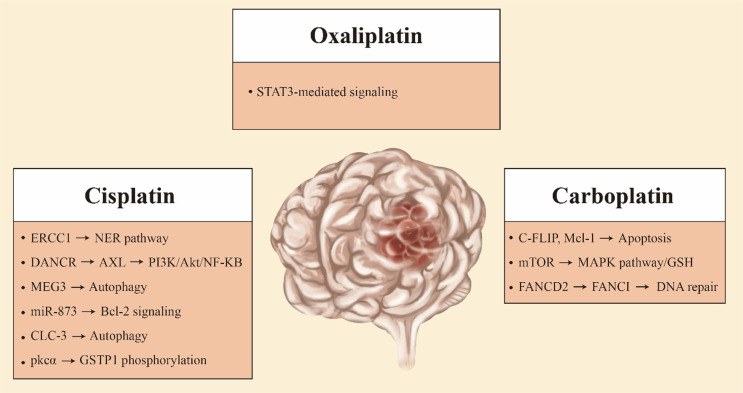
The figure summarizes the resistance mechanisms of platinum anticancer drugs in glioma.

There are many mechanisms involved in the clearance effect of platinum drugs on glioma cells. The drug resistance phenotype of glioma cells to platinum drugs can be generated by changes in any molecule of these mechanisms (Fig. **2**). In addition, the inability of glioma cells to perform the apoptotic procedures normally is also involved in the development of the drug resistance phenotype. We have not found a mechanism that can reasonably explain this phenomenon, but we still reach a consensus in some aspects: (1) drug transport is blocked, leading to the decrease of drug concentration in glioma cells; (2) enhanced ability of glioma cells to inactivate drugs; (3) DNA repair ability is enhanced, and the arrested cell cycle continues to run; (4) apoptotic pathways are blocked and glioma cells cannot be eliminated (Fig. **3**) [16].

### 3.1 Molecular Mechanisms for Cisplatin Resistance in Gliomas

The NER (nucleotide excision repair) is an important protective mechanism in DNA damage caused by ultraviolet rays and chemical molecules. It plays an important role in regulating the resistance of platinum drugs. There is a difference in the expression of excision-repair cross-complementation group 1 (ERCC1) mRNA between non-tumor brain tissues and malignant brain tumor tissues [17]. It has been proven that ERCC1 belongs to the NER pathway and is required for cisplatin damage repair [18]. ERCC1 has been found to be involved in drug resistance in a variety of tumor tissues [19, 20]. Chen *et al.* found that compared with cisplatin-sensitive human glioma tissues, the expression level of ERCC1 was higher in cisplatin-resistant human glioma tissues [21]. Furthermore, they found differences in the methylation levels of the ERCC1 promoter region between cisplatin-resistant human glioma tissues and cisplatin-sensitive human glioma tissues in subsequent studies. This methylation level is inversely correlated with the expression level of ERCC1 [22]. Decreased methylation levels in the ERCC1 promoter region lead to up-regulation of the expression of ERCC1, which in turn leads to the development of glioma resistance to cisplatin treatment.

Long non-coding RNAs (lncRNAs) are a class of non-coding RNAs greater than 200 nt in length, which have important functions in transcriptional silencing, transcriptional activation, chromosomal modification, and intranuclear transport [23, 24]. More and more studies have shown that lncRNAs play an important role in the occurrence, development, and chemoresistance of gliomas [25-27]. The lncRNA HOXD cluster antisense RNA 1 (HOXD-AS1) resulted in cisplatin resistance in glioma cells by binding miR-204 [28]. The lncRNA differentiation antagonizing non-protein coding RNA (DANCR) has been reported to play an oncogenic role in varieties of cancers [29, 30]. DANCR upregulates the expression of AXL in glioma cells, which in turn activates the phosphatidylinositol-3-kinase (PI3K)/Akt/nuclear factor kappa B (NF-κB) signaling pathway. Inhibition of this signaling pathway will improve glioma sensitivity to cisplatin [31, 32]. LncRNA DANCR contributes to drug resistance in glioma cells by activating the AXL/PI3K/Akt/NF-κB signaling pathway [33]. Studies have reported that lncRNA maternally expressed gene3 (MEG3) expression is down-regulated in a variety of tumor cells while exerting tumor-suppressive effects [34, 35]. LncRNA MEG3 was previously reported to be involved in autophagy activation in bladder cancer cells [36]. LncRNA MEG3 was found to be down-regulated in glioma-resistant cell lines. In glioma cells, LncRNA MEG3 also simultaneously reduced cisplatin-induced autophagy [37]. The increased autophagy induced by LncRNA MEG3 improved the chemoresistance of glioma cells to cisplatin.

MicroRNAs (miRNAs) are small endogenous non-coding RNA molecules consisting of approximately 21-25 nucleotides. These miRNAs usually target one or more mRNAs and regulate gene expression by repressing or breaking target mRNAs at the translational level [38]. The expression levels of miRNAs vary in many tumor tissues, including glioma [39-41]. Yue *et al.* found that the content of miR-205 in serum samples from glioma patients was lower than that in normal samples [42]. MiR-205 expression was reduced in cisplatin-resistant glioma cell lines. In addition, miR-205 directly targets E2F transcription factor 1 (E2F1) to downregulate its expression in cisplatin-sensitive glioma cell lines [43]. MiR-205 confers cisplatin resistance in glioma cells by up-regulating the expression level of E2F1. MiR-136 also contributes to cisplatin resistance in glioma cells through a similar mechanism [44]. Overexpression of miR-873 increased apoptosis in cisplatin-resistant glioma cells. In addition, the expression of miR-873 was down-regulated while the expression of B-cell lymphoma 2 (Bcl-2) was up-regulated in glioma tissues compared with normal brain tissues. MiR-873 mediates cisplatin resistance in glioma cells by increasing the protein level of Bcl-2 [45].

Previous studies have reported that autophagy mediates cisplatin resistance in a number of tumor cells [46, 47]. LncRNA MEG3 was reported to be involved in cisplatin resistance in glioma cells through autophagy [37]. Retinoblastoma protein (RB) exerts tumor-suppressive effects by mediating cell cycle arrest [48]. Liu *et al.* found that in glioma cell lines, RB can elevate autophagy to enhance drug resistance [49]. Su *et al.* found that chloride channel 3 (CLC-3) can activate both Akt/mammalian target of the rapamycin (mTOR) signaling pathway and autophagy to mediate drug resistance in glioma cell lines. When the Akt/mTOR pathway is inhibited, chloride channel 3 can still increase resistance by activating autophagy [50].

Glutathione S-transferase pi gene (GSTP1) plays a role in cisplatin resistance in a variety of tumors [51, 52]. GSTP1 is a member of the GST family, which can catalyze the paired binding of endogenous and exogenous compounds and GSH [53]. It was reported that the activation of protein kinase C alpha (PKCα) and subsequent GSTP1 phosphorylation were closely associated with decreased formation of inter-strand pairing cross-links of cisplatin and DNA as well as increased cisplatin resistance [54]. PKCα increases cisplatin metabolism in glioma cell lines by phosphorylating GSTP1 on a serine-dependent basis, leading to drug resistance development [55].

### 3.2 Molecular Mechanisms for Carboplatin Resistance in Gliomas

Carboplatin is a new generation of platinum chemotherapy drugs, with fewer side effects than cisplatin. Carboplatin leads to DNA damage by forming adducts with DNA, which induces apoptosis in tumor cells [56, 57]. mTOR is an important eukaryotic cell signal involved in DNA and protein synthesis transcription, regulating cell growth, metabolism, and apoptosis [58]. When the mTOR signaling pathway is inhibited, the mitogen-activated protein kinase (MAPK) pathway is activated and glioma cells have increased sensitivity to carboplatin. In carboplatin-resistant glioma cell lines, the activated mTOR signaling pathway increased the level of GSH. Activation of the mTOR signaling pathway is one of the causes of carboplatin resistance in pediatric low-grade glioma [59].

When glioma cells were treated with carboplatin, the protein levels of cellular FLICE-inhibitory protein (c-FLIP) and myeloid cell leukemia 1 (Mcl-1) were decreased, while apoptosis was inhibited. But the expression of c-FLIP and Mcl-1 was upregulated in carboplatin-resistant glioma cell lines. C-FLIP and Mcl-1 are involved in carboplatin resistance in glioma cells [60]. Recent studies have shown that Fanconi anemia group D2 protein (FANCD2) can modulate the effect of carboplatin therapy in children with high-grade gliomas [61]. FANCD2 can bind to FA complementation group I (FANCI) to participate in the repair of DNA damage [62]. In glioma cell lines resistant to carboplatin, the sensitivity of FANCD2 to repair damaged DNA formation is enhanced [61-63].

### 3.3 Molecular Mechanisms for Oxaliplatin Resistance in Gliomas

Oxaliplatin is a third-generation platinum drug that is involved in regulating DNA replication and transcription as well as in regulating tumor immunity [64-66]. Oxaliplatin has a relatively strong antitumor effect on glioma cells. Upregulated expression of signal transducer and activator of transcription 3 (STAT3) was found in glioma cell lines resistant to oxaliplatin [67]. Studies indicated that STAT3-mediated signaling played an important role in the resistance of glioma cells to oxaliplatin [65, 67].

## CONCLUSION

Platinum drugs are often used clinically for the treatment of low-grade gliomas [10, 13]. The rapid generation of drug resistance of glioma cells often leads to the failure of platinum drug therapy. As described in this paper, there are at least four mechanisms involved in the resistance of glioma cells to platinum drugs. At present, there is no literature showing that a certain mechanism plays a leading role in it. We speculate that a variety of mechanisms may be involved in the emergence of this therapeutic resistance. These mechanisms with spatial heterogeneity may work at the same time. Previous studies have shown that the inhibition of a certain signaling pathway cannot completely reverse this drug resistance phenotype. Recently discovered molecules and therapies often change the sensitivity of glioma cells to platinum drugs by inhibiting multiple signaling pathways. For example, knockdown of miR-106a can inhibit the expression of P-glycoprotein (P-gp) and multidrug resistance-associated protein (MRP) and significantly improve the sensitivity of glioma cells to cisplatin. Knockdown of miR-106a can also improve the effectiveness of treatment by inhibiting the expression of GST-π and ERCC1. This means that miR-106a is an effective target to reverse the resistance of gliomas to platinum drugs treatment [68]. In addition, interleukin-24 (IL-24) in glioma cells affects the sensitivity of gliomas to cisplatin by regulating the expression levels of P-gp and Bcl-2. IL-24 may be a biomarker predicting the sensitivity of gliomas to chemotherapy [69]. Peroxisome proliferator-activated receptor-γ (PPAR-γ) also plays an important role in regulating the sensitivity of glioma cells to cisplatin. When the translation of PPAR-γ in glioma cells increases, the expression levels of multidrug resistance mutation 1 (MDR1) and multidrug resistance-associated protein 1 (MRP1) genes decrease, leading to the accumulation of cisplatin in glioma cells. In addition, overexpression of PPAR-γ can inhibit the expression of GST-π and GSH, thereby interfering with the role of the GSH system in the drug resistance of glioma cells. PPAR-γ can also induce tumor cell apoptosis by positively regulating the expression of P53 [70]. Cytokine-induced killer cells (CIK) therapy is a brand-new method of tumor treatment. When cisplatin-resistant U87 cells interact with CIK, the expressions of MDR-1, MRP-1, GST-π, and Bcl-2 are all down-regulated, thus reversing the drug resistance of gliomas. Based on these results, CIK therapy is expected to play a positive role in improving the chemotherapy resistance of glioma patients [71].

An efficient drug delivery system creates extremely high concentrations of drugs locally in the tumor, which minimizes the side effects of the drug to increase its efficacy [72, 73]. Currently, liposomes are one of the most mature drug delivery platforms for platinum drug delivery [74]. Lipusu, L-NDDP and SPI-77 are the liposomes that have been developed so far. Clinical trial results show that these liposomes are not only effective in delivering cisplatin but also have little toxicity [75-77]. In addition, nanotechnology plays an important role in improving drug delivery efficiency and reducing side effects [78, 79]. In animal experiments, the efficacy of oxaliplatin can be improved by using hyaluronic acid to polymerize nanoparticles [80]. Magnetic resonance-guided focused ultrasound systems can open the blood-brain barrier in a short time by using microbubbles. This technique can improve the efficiency of drug delivery to the central nervous system [81]. In a mouse glioma model, this technique enhanced carboplatin delivery, reduced tumor growth and improved survival [82].

Based on the above findings, it is reasonable to believe that avoiding at least two mechanisms of drug resistance can restore the sensitivity level of glioma cells to platinum drugs to effective therapeutic concentrations. However, the current dilemma is which mechanism of drug resistance should be blocked first in order to achieve the best therapeutic effect. This may be related to the type and pathological grade of gliomas. We boldly speculate: if we can simultaneously block the mechanisms that occur in the cell membrane, cytoplasm, and nucleus, then it may be possible to restore the sensitivity of glioma cells to platinum drugs treatment. The discovery of new chemo-sensitization regimens and biomarkers for chemosensitivity prediction is helped by understanding the mechanisms of platinum drug resistance in gliomas. These, combined with the glioma classification tools in the hands of clinicians can better classify patients, and ultimately develop efficient chemotherapy regimens with fewer side effects.
